# Proteomic profile at the time of surgery correlates with disease stage and surgical outcome in periprosthetic joint infection

**DOI:** 10.1128/mbio.01700-25

**Published:** 2025-08-28

**Authors:** Kathleen O’Connor, Christina Koscianski, Nicholas Larson, Kiran K. Mangalaparthi, Cody Hoffmann, Nicholas A. Bedard, Khaled Elmenawi, Merrick T. Ducharme, Jessica D. Hohenstein, Daniel O’Brien, Akhilesh Pandey, Robin Patel

**Affiliations:** 1 Division of Clinical Microbiology, Mayo Clinic 315212, Rochester, Minnesota, USA; 2 Department of Quantitative Health Sciences Research, Mayo Clinic 195006 , Rochester, Minnesota, USA; 3 Department of Laboratory Medicine and Pathology, Mayo Clinic 195112 , Rochester, Minnesota, USA; 4 Department of Orthopedic Surgery, Mayo Clinic 314208, Rochester, Minnesota, USA; 5 Center for Individualized Medicine, Mayo Clinic 315292 , Rochester, Minnesota, USA; 6 Manipal Academy of Higher Education 76793, Manipal, Karnataka, India; 7 Division of Public Health, Infectious Diseases, and Occupational Medicine, Mayo Clinic 314201, Rochester, Minnesota, USA; The University of Mississippi Medical Center, Jackson, Mississippi, USA

**Keywords:** acute infection, chronic infection, prosthetic joint infection, proteomics, immunology

## Abstract

Periprosthetic joint infection (PJI) is the most common and difficult to treat form of arthroplasty failure. While treatment with debridement, antibiotics, and implant retention (DAIR) is preferable to one- or two-stage implant exchange based on morbidity and cost, outcomes are not successful in all cases selected for this management strategy. DAIR is currently recommended when infection is perceived to be in an “acute” phase, based on symptom duration; despite this selection strategy, DAIR failure rates are high. A more objective means of predicting DAIR success could improve patient outcomes by limiting DAIR failures. To explore this possibility, advanced proteomic analysis of sonicate fluid samples from 95 subjects with PJI was performed using proximity extension assay (PEA), with 77 samples additionally undergoing complementary proteomics analysis with liquid chromatography with tandem mass spectrometry (LC-MS/MS). Sparse partial least squares differentiation analysis (sPLS-DA) separated samples from patients who underwent DAIR with known outcomes (*n* = 19) into successful or unsuccessful groups, predicting DAIR surgical outcome (area under the curve [AUC]: 1; mean “leave one out” AUC: 0.6). Proteins predictive of DAIR surgical outcome were significantly enriched for the “protein repair” Gene Ontology (GO) pathway (*P*-adj = 0.02). Additionally, proteomic signatures associated with symptom duration in PJI categorized samples into short and long symptom duration PJI. In summary, an investigation of 7,261 unique human proteins separated PJI-associated samples based on symptom duration and identified a proteomic profile predictive of DAIR success.

**IMPORTANCE:**

Chronic infections are generally understood to last months to years, with acute infections lasting days to weeks. The transition from acute to chronic infection is, however, poorly understood. Periprosthetic joint infection (PJI) has been considered more challenging to treat when “chronic” than when “acute.” A surgery preferred for its recovery time and cost—debridement, antibiotics, and implant retention (DAIR)—is recommended for PJI management when symptom duration is short; yet, even in this select patient group, DAIR is associated with a high treatment failure rate. A means to better identify those predicted to have a successful outcome if they undergo DAIR is needed. Here, over 7,000 proteins from human clinical PJI samples were measured and shown to be able to separate samples based on symptom duration. Importantly, a proteomic profile predictive of DAIR success was identified.

## INTRODUCTION

Chronic diseases are becoming more common as treatments for once-fatal acute conditions are developed and as the population ages (1). The U.S. healthcare system is said to work best for acute diseases (1), with chronic diseases being more challenging. Chronic diseases are generally understood to be persistent and recurrent, with duration measured in months to years, rather than days to weeks (2–6). Chronic bacterial infections remain an understudied area despite their impact. While the true extent of chronic bacterial infections has not been defined, 2% of the US population is affected by chronic wounds alone (7). Including infections such as indwelling device infections (8), chronic lung infections (9, 10), and recurrent urinary tract infections (11), would increase the number affected to 10s of millions in the US. Despite the prevalence of chronic infections, how they differ from acute infections and their immunological underpinnings is poorly understood.

Periprosthetic joint infection (PJI) is a devastating complication of total joint arthroplasty that requires surgery for successful treatment (12–16). Today, the surgical approach is often based on perceived acuity, with different procedures utilized if PJI is considered to be in an “acute” or “chronic” stage. With the latter, implant removal is performed as a one- or two-stage exchange (12). Conversely, the former may be treated with debridement, antibiotics, and implant retention (DAIR) (17–21), a procedure with less morbidity and cost than implant removal (22, 23). From both a patient and surgeon perspective, DAIR is a preferred PJI treatment strategy, but only if the outcome is successful, as failed DAIR requires additional surgery. Conversely, unnecessary implant removal (i.e., two-stage exchange performed when DAIR would have been successful) results in additional morbidity. Thus, there is a need for an accurate means of predicting the successful outcome of DAIR.

Implant resection is theoretically preferred for “chronic” PJI because of the presence of mature biofilm, necessitating a more aggressive approach with implant removal. Despite general agreement in the field that DAIR is best performed on “acute PJI,” the timing of disease stage transition from “acute” to “chronic” and how to determine duration of infection is debated (24). There is no single accepted definition for “acute” versus “chronic” PJI, and perhaps consequently, DAIR surgery success rates range from 50% to 95% (25–35), despite targeting “acute” cases. Zimmerli et al. suggest that DAIR is appropriate for early post-operative PJI or acute hematogenous PJI (36); yet, DAIR procedures may fail due to persistent infection despite being performed in these scenarios and may be successful even when performed outside of these situations (20, 25, 37–42). Definitions of “acute” PJI are often based on time between surgery and symptom onset (2–12 weeks [20, 37–39]), or duration of symptoms (2–3 days to 3 weeks [20, 25, 40–42]), with inconsistent timings reported. Wide-ranging definitions of “acute” PJI and suboptimal DAIR success rates highlight a need for more objective measures, rather than relying on timing of symptom onset and/or symptom duration, to guide surgical decision-making for PJI.

Sonication of resected arthroplasty devices to generate “sonicate fluid” is an innovative technique that has improved the microbiologic diagnosis of PJI (43). During DAIR or resection surgery, resected arthroplasty components are sonicated in a solution to remove biofilms from implant surfaces and disaggregate them into sonicate fluid, a process that also samples host and microbial proteins. Here, the host proteome of sonicate fluid from PJI was investigated to attempt to define a signature for symptom duration and surgical outcomes.

This study was approached with the hypothesis that there is not a specific time at which PJI transitions from being “DAIR-treatable” to “DAIR-untreatable,” but rather that this depends on infection physiology specific to each patient and infecting microorganism. The host proteome at the site of PJI has the potential to reveal signatures predictive of DAIR success. Ultimately, findings might be used to design a clinically useful panel test to inform the selection of surgical strategy. To identify biomarkers for potential use as DAIR-success predictive tests, the proteomic signature at the surface of PJI implants was accessed using two complementary platforms—liquid chromatography with tandem mass spectrometry (LC-MS/MS) and the Explore 3072 platform based on proximity extension assay (PEA). The study revealed differential proteomic-based physiology of short and long symptom duration PJI; additionally, where commonly performed laboratory tests, patient characteristics, age, or patient-reported symptom duration failed to predict DAIR outcomes, a multivariate proteomic model of 120 proteins predicted DAIR outcomes post hoc.

## RESULTS

### C-reactive protein is elevated in short-duration PJI

Symptom duration was sorted into short (<4 weeks, 19 samples), intermediate (4–12 weeks, 31 samples), and long (>12 weeks, 37 samples) duration groups, and an unsorted group (missing symptom duration data), and demographics and commonly collected pre-surgical laboratory tests compared between groups (Table 1). There were no significant differences between subject age, the presence of a sinus tract, sex, race, synovial fluid neutrophil percentage, or synovial fluid cell count between the groups. Mean C-reactive protein (CRP) (mg/dL) was over twofold higher in the short compared to the long or intermediate symptom duration groups, and CRP was significantly different between short and intermediate, short and long, and short and unsorted symptom duration groups.

**TABLE 1:** Demographic and pre-surgical laboratory data from 95 PJI patients^*a*^

Parameter	Short symptom duration (*n* = 19)		Intermediate symptom duration (*n* = 31)		Long symptom duration (*n* = 37)		Unsorted symptom duration (*n* = 8)	
	Mean	Standard deviation	Mean	Standard deviation	Mean	Standard deviation	Mean	Standard deviation
Mean symptom duration (weeks)	1.5	0.9	8.3	3.1	48.9	66.9	NA	NA
Mean ESR (mm/hour)	58.2	32.6	56.1	28.5	47.1	29.8	54.9	31.0
Mean CRP (mg/dL)	136.0	91.1	59.4	56.8	37.0	37.3	37.4	33.5
Mean synovial fluid neutrophil (%)	88.2	17.6	91.6	6.3	87.5	7.7	96.0	1.6
Mean synovial fluid nucleated cell count (cells/mL)	74,467.4	63,988.0	51,018.8	33,007.6	46,703.0	43,751.9	20,863.0	13,734.8
Sinus tract present (%)	21.1	41.9	16.1	37.4	5.4	22.9	12.5	35.4
Female (%)	36.8	49.6	38.7	49.5	44.4	50.4	50.0	53.5
Mean age (years)	65.7	14.4	66.1	15.7	65.7	15.4	62.9	8.3
Laterality: right (%)	36.8	50.7	38.7	48.6	44.4	50.7	50.0	53.5
Race: White (%)	89.5	0.3	93.5	0.2	100.0	0.0	87.5	0.4

^*a*^ C-reactive protein (CRP, mg/dL) was significantly different between the short and intermediate symptom duration groups (*P* value < 0.001), between the short and long symptom duration groups (*P* value < 0.001), and between the short and unsorted symptom duration groups (*P* value < 0.001). Other differences between groups were not statistically significant. ESR, erythrocyte sedimentation rate.

### Univariate analysis reveals proteomic differences in sonicate fluid based on symptom duration

**Fig 1:**
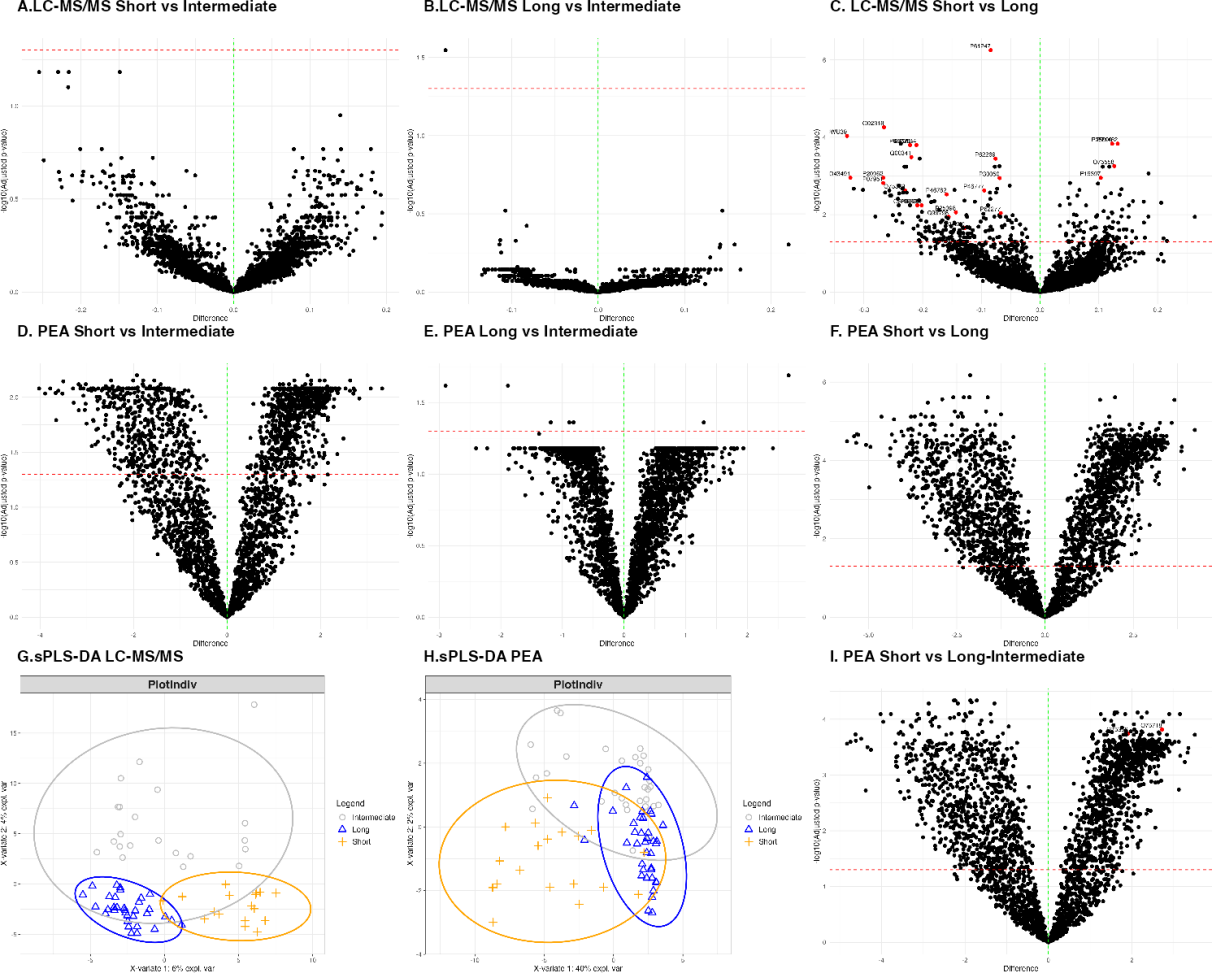
Univariate and multivariate analyses reveal proteomic differences between short and long symptom duration PJI. t-Tests were performed to compare the LC-MS/MS and PEA sequenced proteins for the short versus long, short versus intermediate, and long versus intermediate symptom duration groups; false discovery rate (FDR)-adjusted *P* values are plotted. For LC-MS/MS, no proteins were differentially expressed between short versus intermediate groups (A), with one protein significantly differentially expressed between long versus intermediate symptom duration groups (1/5,794, 0.02%) (B). There were 275/5,794 (4.75%) proteins differentially expressed between short and long symptom duration groups (C). AUC, specificity, and sensitivity were calculated for the short versus long symptom duration group; the 39/5,794 (0.67%) values that met the criterion of AUC ≥0.9 are shown in red. When calculating FDR-adjusted *P* values for PEA data, there was a different pattern. Long versus intermediate symptom duration groups had few significantly differentially expressed proteins (7/2,888, 0.25%) (D), whereas there were many significant proteins differentially expressed between short versus intermediate (1,286/2,888, 44%) (E) and short versus long (2,024/2,888, 70%) (F) symptom duration groups. Multivariate analysis was performed using sparse partial least squares differentiation analysis (sPLS-DA) to differentiate short, intermediate, and long symptom duration groups. LC-MS/MS data showed good sorting of short from long symptom duration groups down component 1, with the intermediate symptom duration group appearing to cluster with either the short or long group by component 1 (mean model AUCs—intermediate: 0.41, short: 0.88, long: 0.788) (G). PEA data also appeared to separate short and long symptom duration groups by component 1, although the intermediate symptom duration group appeared to cluster mostly with the long symptom duration group, rather than spreading across short and long symptom duration groups evenly (H). Therefore, for PEA data, long and intermediate symptom duration groups were grouped together into a combined long-intermediate symptom duration group and compared to the short symptom duration group for t-test analysis (I) (1,852/2,888 [64%] significant proteins, 2/2,888 [0.06%] proteins AUC ≤ 0.9), with an AUC of ≥0.9 are shown in red.

Proteins (275/5,794, 4.75%) were significantly differentially abundant between short and long symptom duration, but not between short and intermediate symptom duration groups, with a single protein (1/5,794, 0.02%) difference between intermediate and long symptom duration groups (Fig. 1A through C; Table S1). Area under the curve (AUC) for the short versus long group was calculated; 39/5,794 (0.67%) proteins had an AUC ≥0.9. For PEA data, long versus intermediate symptom duration groups had few significantly differentially expressed proteins (7/2,888, 0.24%, Fig. 1D)**,** whereas there were many differentially abundant proteins between the short and intermediate (1,286/2,888, 44%, Fig. 1E)**,** and short and long (2,024/2,888, 70%, Fig. 1F) symptom duration groups. Spearman rank tests were performed on the PEA and LC-MS/MS data. The significance of each protein was tested by patient-reported symptom duration in weeks, excluding unsorted samples. A total of 301/5,794 (5%) proteins were significant for LC-MS/MS Spearman, and 2,042/2,888 (70%) for PEA Spearman (Tables S3 and S4). Given that LC-MS/MS and PEA data generated different clustering results, overlapping proteins were assessed. There were 1,421 proteins measured by both techniques, of 2,888 proteins covered by PEA and 5,794 proteins by LC-MS/MS; Spearman rank correlation was used to compare results. A total of 356/1,421 (25%) proteins were significantly positively correlated. A small subset of proteins (32/1,421 [2%]) was significantly negatively correlated, meaning they were upregulated in samples analyzed by one technique and downregulated in the same samples analyzed using the other technique. The fold change of all proteins for LC-MS/MS (short versus long symptom duration) against PEA (short versus long symptom duration) was plotted using symptom duration data, including Spearman rank data (Fig. S1).

### Multivariate analysis clusters samples by symptom duration differently based on proteomic technique

The mixOmics package was used to perform sparse partial least squares differentiation analysis (sPLS-DA) on PEA and LC-MS/MS data sets to differentiate short, intermediate, and long symptom duration groups, excluding unsorted samples. Short and long groups separated well down component 1 for LC-MS/MS data, with the intermediate group appearing to cluster with both short and long symptom duration groups by component 1; 40/5,794 (0.7%) proteins contributed to component 1 (Fig. 1G; Table S4). mFold validation performed to determine mean AUCs for the models determined that the intermediate (mean AUC 0.41) compared to short and long symptom duration groups was poorly sorted (mean AUCs: 0.88, 0.788, respectively). PEA data also separated the short and long symptom duration groups along component 1, although the intermediate symptom duration group clustered mostly with the long symptom duration group, rather than spreading evenly across the short and long symptom duration groups; 30/2,888 (1%) proteins contributed to component 1 (Fig. 1H; Table S5). mFold validation again showed that the intermediate symptom duration group was poorly sorted (intermediate mean AUC: 0.44, short mean AUC: 0.83, long mean AUC: 0.73). The multivariate and t-test analyses above suggest that LC-MS/MS has two types of “intermediate” protein signatures—half more like the short, and the other half more like the long symptom duration group. However, for PEA, the intermediate clustered best with the long symptom duration group and was distinct from the short symptom duration group. Therefore, long and intermediate symptom duration groups were combined for PEA data into a “long-intermediate symptom duration” group for downstream analyses. The combined long-intermediate was compared with the short symptom duration group for t-test analyses; 1,852/2,888 (64%) significant proteins were found that met criteria for AUC ≥0.9 (2/2,888, 0.07%, Fig. 1I; Table S6).

### LC-MS/MS identifies an acute immune response profile in short symptom duration PJI

**Fig 2:**
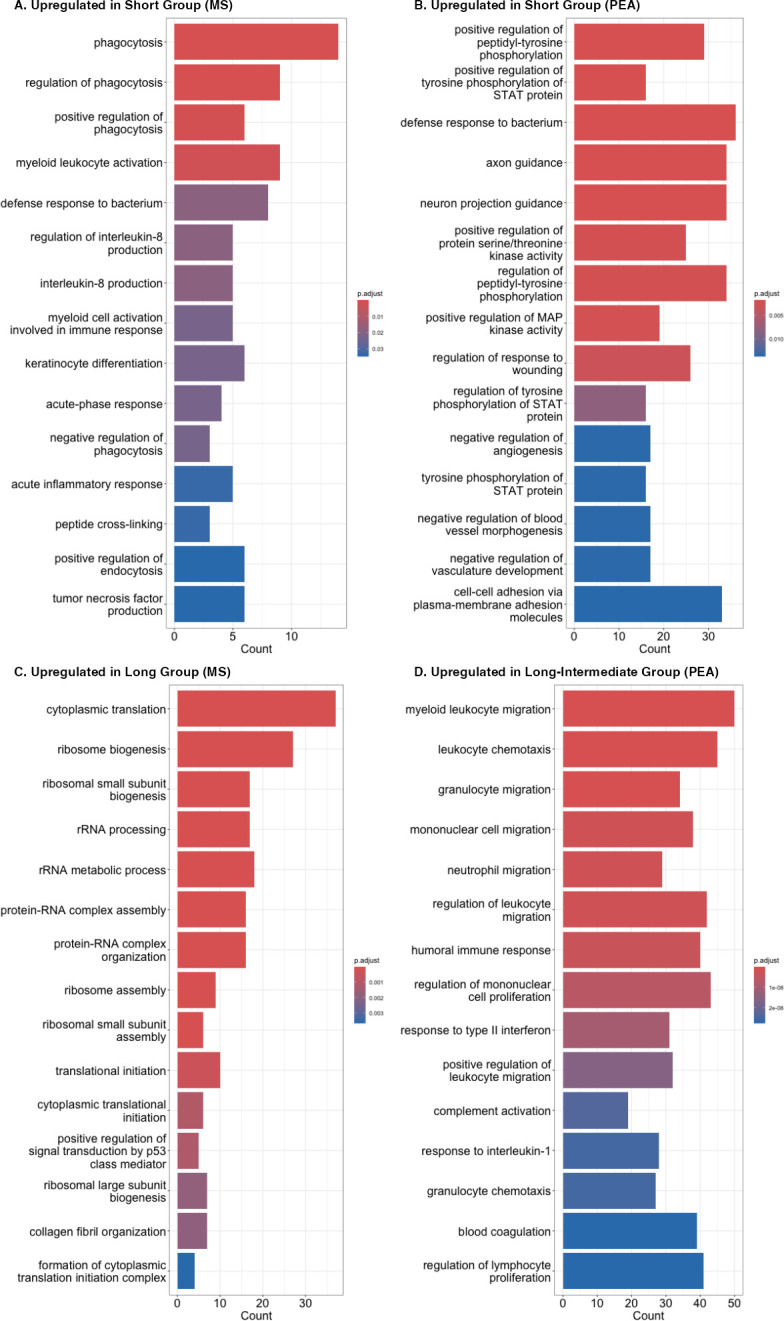
Functionally enriched Gene Ontology (GO) pathways for short and long symptom duration PJI. Enrichment of functional GO pathways was determined for proteins significantly up- or down-regulated (based on *t*-test). Queries were performed by proteomic analysis type, and whether proteins were up- or down-regulated. Functional groups upregulated in the short symptom duration group were compared to the long symptom duration group for LC-MS/MS (A). Similarly, groups upregulated in the short symptom duration group were compared to the long-intermediate symptom duration group for PEA (B). Protein functional groups upregulated in the long symptom duration group were determined for the LC-MS/MS data (C). Finally, protein functional groups upregulated in the long-intermediate symptom duration group were determined for PEA data (D).

Using proteins identified by the univariate *t*-test analyses, PEA and LC-MS/MS identified different groups of protein function as upregulated in the short symptom duration group (Fig. 2). LC-MS/MS identified multiple GO pathways as upregulated with functions relating to phagocytosis, including “phagocytosis,” “regulation of phagocytosis,” “positive regulation of phagocytosis,” “negative regulation of phagocytosis,” and related to phagocytosis, “positive regulation of endocytosis.” “Defense response to bacterium” was among the most significant upregulated functions after phagocytosis-related processes. Two “acute” functions (based on labeling) were upregulated in the short duration group, including “acute-phase response” and “acute inflammatory response,” suggesting an association of short symptom duration with an acute phase of PJI (Fig. 2A).

### PEA identified downregulation of angiogenesis as a functional category upregulated in short symptom duration PJI

Similar to LC-MS/MS, PEA identified “defense response to bacterium” as upregulated in the short compared to the combined long-intermediate symptom duration group. PEA also identified “axon guidance” and “neuron projection guidance” as upregulated in the short symptom duration group, suggesting that the nervous system may play a role in acute infection. Multiple protein functions having to do with STAT protein, involved in angiogenesis, were upregulated in the short symptom duration group, including “tyrosine phosphorylation of STAT protein,” “positive regulation of tyrosine phosphorylation of STAT protein”, “positive regulation of peptidyl-tyrosine phosphorylation” and “regulation of peptidyl-tyrosine phosphorylation.” There were multiple functions having to do with downregulation of angiogenesis, including “negative regulation of blood vessel morphogenesis,” “negative regulation of angiogenesis,” and “negative regulation of vasculature development,” which may have implications for blood flow during PJI. Kinase activity was impacted, with “positive regulation of MAP kinase activity” and “positive regulation of serine/threonine kinase activity” upregulated in the short symptom duration group.

### GO enrichment: PEA and LC-MS/MS identify different functions as upregulated in the long symptom duration group

LC-MS/MS primarily identified functions relating to translation as upregulated in the long symptom duration group, including multiple ribosomal, rRNA, and protein-RNA functions. Peptidase and endopeptidase functions, as well as cellular respiration, were also upregulated in this group (Fig. 2C). PEA identified multiple functions having to do with leukocyte movement, specifically “migration” and “chemotaxis,” as upregulated in the long-intermediate symptom duration group. Also upregulated were the proliferation of lymphocytes and mononuclear cells. Relating to well-studied aspects of the immune response, “humoral immune response,” “complement activation,” “blood coagulation,” “response to interleukin-1,” and “response to interferon-γ” were upregulated in the long-intermediate compared to the short symptom duration group. Upregulation of these functions in the long-intermediate symptom duration group suggests a delay in their activation as part of the immune response during PJI (Fig. 2D).

### Hierarchical clustering sorts PJI samples into stage 1 and stage 2 PJI

**Fig 3:**
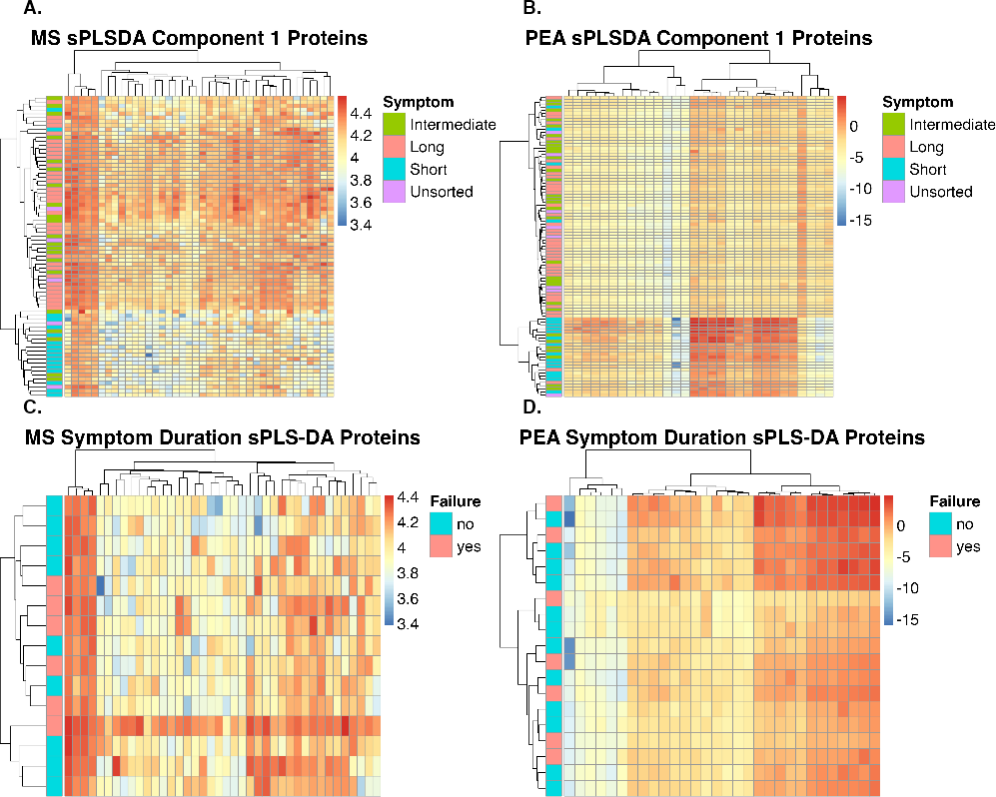
Hierarchical clustering of samples by symptom duration or DAIR outcome using sPLS-DA model-identified proteins. Proteins determined by sPLS-DA models to display protein variation between samples of different symptom durations were determined. LC-MS/MS (**A**) and PEA (**B**) data showed similar patterns. Using hierarchical sorting, the samples were sorted into two primary groups: one group with mostly short symptom duration samples, labeled the “stage 1 PJI” group, and a second group with a majority of long symptom duration samples, labeled as “stage 2 PJI” group. Intermediate samples were split between stage 1 and stage 2 groups, with a majority sorting with long symptom duration samples. Unsorted symptom duration samples were also split between stage 1 and stage 2 groups. The same proteins from panels A and B were used to sort DAIR surgery samples by failure (C and D). DAIR samples did not form clusters by surgical outcome based on sPLS-DA identified proteins.

Using collections of proteins found to be important by sPLS-DA, hierarchical clustering of the PJI samples was performed for the LC-MS/MS and PEA data sets. Both hierarchical clustering models grouped samples into two primary groups, one with the shortest symptom duration samples (“stage 1 PJI” group), and another with the longest symptom duration samples (“stage 2 PJI” group). The sorting is shown as heatmaps in Fig. 3A and B, with samples binned into the two stages in Table 2. A majority, 15/19 (79%), of short duration samples were categorized as stage 1 by both metrics. The intermediate symptom duration group had 6/31 (19%) samples categorized as stage 1 by both metrics, 1/31 (3%) categorized as stage 1 by one metric and stage 2 by the other, and 22/31 (70%) categorized as stage 2 by both metrics. A total of 35/37 (95%) of long symptom duration samples were categorized as stage 2 by all tested metrics (either 1/1 or 2/2 assays).

**TABLE 2:** Protein expression data and hierarchical sorting as determined using the pheatmap package^*a*^

Sample name	DAIR failure (yes/no)	Symptom duration (weeks)	Sample type	Label	LC-MS/MS sPLSDA	PEA sPLSDA
CK47	No	0.42	*Streptococcus pyogenes*	Short	Stage 1 PJI	Stage 2 PJI
CK54	Yes	0.42	*Staphylococcus aureus*	Short	Stage 1 PJI	Stage 1 PJI
CK69		0.42	*Streptococcus agalactiae*	Short	Stage 1 PJI	Stage 1 PJI
CK82	No	0.57	*S. agalactiae*	Short	Stage 1 PJI	Stage 1 PJI
CK34	No	0.71	*Morganella morganii*	Short	Stage 1 PJI	Stage 1 PJI
CK65	No	0.71	*Staphylococcus lugdunensis*	Short	Stage 1 PJI	Stage 1 PJI
CK116		1	*Staphylococcus capitis*	Short	NA	Stage 2 PJI
CK12	No	1	*S. lugdunensis*	Short	Stage 1 PJI	Stage 1 PJI
CK71	Yes	1	*Enterococcus faecalis*	Short	Stage 1 PJI	Stage 1 PJI
CK87		1	*S. aureus*	Short	Stage 1 PJI	Stage 1 PJI
CK5	Yes	1.42	*S. aureus*	Short	Stage 1 PJI	Stage 1 PJI
CK17	No	2	*S. aureus*	Short	Stage 1 PJI	Stage 1 PJI
CK40		2	*E. faecalis*	Short	Stage 1 PJI	Stage 1 PJI
CK59		2	*Staphylococcus caprae*	Short	Stage 2 PJI	Stage 2 PJI
CK67		2	*Staphylococcus epidermidis*	Short	Stage 1 PJI	Stage 1 PJI
CK86		2	*S. aureus*	Short	Stage 2 PJI	Stage 2 PJI
CK113	No	2.71	*Citrobacter koseri*	Short	NA	Stage 1 PJI
CK66	No	3	*S. epidermidis*	Short	Stage 1 PJI	Stage 1 PJI
CK21	No	3.42	*S. lugdunensis*	Short	Stage 1 PJI	Stage 1 PJI
CK107	Yes	4	*Clostridium ramosum*	Intermediate	NA	Stage 1 PJI
CK112		4	*Abiotrophia defectiva*	Intermediate	NA	Stage 2 PJI
CK124		4	*Candida albicans*	Intermediate	NA	Stage 1 PJI
CK14	No	4	*S. epidermidis*	Intermediate	Stage 1 PJI	Stage 1 PJI
CK16		4	*Streptococcus dysgalactiae*	Intermediate	Stage 2 PJI	Stage 2 PJI
CK52		4	*S. aureus*	Intermediate	Stage 2 PJI	Stage 2 PJI
CK70		4	*S. aureus*	Intermediate	Stage 2 PJI	Stage 2 PJI
CK57	Yes	5	*S. epidermidis*	Intermediate	Stage 2 PJI	Stage 2 PJI
CK78		5	*Granulicatella adiacens*	Intermediate	Stage 2 PJI	Stage 2 PJI
CK119		6	*Haemophilus parainfluenzae*	Intermediate	NA	Stage 2 PJI
CK1		8	*S. epidermidis*	Intermediate	Stage 2 PJI	Stage 2 PJI
CK117	No	8	*Corynebacterium striatum*	Intermediate	NA	Stage 2 PJI
CK123		8	*S. epidermidis*	Intermediate	NA	Stage 2 PJI
CK127	No	8	*Finegoldia magna*	Intermediate	NA	Stage 1 PJI
CK13		8	*E. faecalis*	Intermediate	Stage 2 PJI	Stage 2 PJI
CK19		8	*S. epidermidis*	Intermediate	Stage 2 PJI	Stage 2 PJI
CK72		8	*Enterococcus faecium*	Intermediate	Stage 2 PJI	Stage 2 PJI
CK55	Yes	9	*S. epidermidis*	Intermediate	Stage 1 PJI	Stage 1 PJI
CK8		9	*S. epidermidis*	Intermediate	Stage 2 PJI	Stage 2 PJI
CK25		10	*S. aureus*	Intermediate	Stage 1 PJI	Stage 2 PJI
CK63		10	*Streptococcus pneumoniae*	Intermediate	Stage 2 PJI	Stage 2 PJI
CK64		11	*S. aureus*	Intermediate	Stage 2 PJI	Stage 2 PJI
CK125		12	*S. epidermidis*	Intermediate	NA	Stage 2 PJI
CK126		12	*Bacteroides fragilis*	Intermediate	NA	Stage 2 PJI
CK15		12	*S. aureus*	Intermediate	Stage 2 PJI	Stage 2 PJI
CK3		12	*Cutibacterium acnes*	Intermediate	Stage 2 PJI	Stage 2 PJI
CK39		12	*S. epidermidis*	Intermediate	Stage 2 PJI	Stage 2 PJI
CK77		12	*E. faecalis*	Intermediate	Stage 1 PJI	Stage 1 PJI
CK79		12	*S. epidermidis*	Intermediate	NA	Stage 2 PJI
CK81		12	*S. aureus*	Intermediate	Stage 2 PJI	Stage 2 PJI
CK9	Yes	12	*Escherichia coli*	Intermediate	Stage 1 PJI	Stage 1 PJI
CK11		14	*Pseudomonas aeruginosa*	Long	Stage 2 PJI	Stage 2 PJI
CK23		14	*S. lugdunensis*	Long	Stage 2 PJI	Stage 2 PJI
CK51		14	*S. agalactiae*	Long	Stage 2 PJI	Stage 2 PJI
CK85		14	*S. agalactiae*	Long	Stage 2 PJI	Stage 2 PJI
CK2		15	*S. epidermidis*	Long	Stage 2 PJI	Stage 2 PJI
CK61		15	*S. aureus*	Long	Stage 2 PJI	Stage 2 PJI
CK26		16	*E. faecalis*	Long	Stage 2 PJI	Stage 2 PJI
CK50		16	*S. epidermidis*	Long	Stage 2 PJI	Stage 2 PJI
CK56		16	*S. aureus*	Long	Stage 2 PJI	Stage 2 PJI
CK43		20	*S. epidermidis*	Long	Stage 2 PJI	Stage 2 PJI
CK110		24	*S. aureus*	Long	NA	Stage 2 PJI
CK36		24	*S. epidermidis*	Long	Stage 2 PJI	Stage 2 PJI
CK45		24	*S. capitis*	Long	Stage 2 PJI	Stage 2 PJI
CK48		24	*C. acnes*	Long	Stage 2 PJI	Stage 2 PJI
CK60		24	*Enterococcus faecalis*	Long	Stage 2 PJI	Stage 2 PJI
CK68		24	*S. epidermidis*	Long	Stage 2 PJI	Stage 2 PJI
CK41		30	*S. epidermidis*	Long	Stage 2 PJI	Stage 2 PJI
CK30		32	*S. epidermidis*	Long	Stage 2 PJI	Stage 2 PJI
CK4		32	*S. epidermidis*	Long	Stage 2 PJI	Stage 2 PJI
CK42		32	*E. faecalis*	Long	Stage 2 PJI	Stage 2 PJI
CK53		32	*S. epidermidis*	Long	Stage 2 PJI	Stage 2 PJI
CK7		33	*S. epidermidis*	Long	Stage 2 PJI	Stage 2 PJI
CK134		35	*S. epidermidis*	Long	NA	Stage 2 PJI
CK62		36	*P. aeruginosa*	Long	Stage 2 PJI	Stage 1 PJI
CK27		38	*S. epidermidis*	Long	Stage 2 PJI	Stage 2 PJI
CK35		40	*E. faecalis*	Long	Stage 2 PJI	Stage 2 PJI
CK38		52	*S. aureus*	Long	Stage 2 PJI	Stage 2 PJI
CK58		52	*E. faecalis*	Long	Stage 2 PJI	Stage 2 PJI
CK83		52	*S. aureus*	Long	Stage 2 PJI	Stage 2 PJI
CK31		59	*S. epidermidis*	Long	Stage 2 PJI	Stage 2 PJI
CK73		62	*S. epidermidis*	Long	Stage 2 PJI	Stage 2 PJI
CK33		64	*S. caprae*	Long	Stage 2 PJI	Stage 2 PJI
CK76		64	*S. epidermidis*	Long	Stage 2 PJI	Stage 2 PJI
CK109		72	*Serratia marcescens*	Long	NA	Stage 1 PJI
CK37		97	*S. aureus*	Long	Stage 2 PJI	Stage 2 PJI
CK106		208	*Corynebacterium jeikeium*	Long	NA	Stage 2 PJI
CK6		388	*S. caprae*	Long	Stage 2 PJI	Stage 2 PJI
CK10		Unknown	*S. epidermidis*	Unsorted	Stage 1 PJI	Stage 2 PJI
CK118		Unknown	*P. aeruginosa*	Unsorted	NA	Stage 2 PJI
CK121		Unknown	*S. lugdunensis*	Unsorted	NA	Stage 2 PJI
CK18		Unknown	*S. epidermidis*	Unsorted	Stage 2 PJI	Stage 2 PJI
CK29		Unknown	*S. aureus*	Unsorted	Stage 2 PJI	Stage 2 PJI
CK74		Unknown	*S. lugdunensis*	Unsorted	Stage 1 PJI	Stage 1 PJI
CK75		Unknown	*S. epidermidis*	Unsorted	NA	Stage 2 PJI
CK84		Unknown	*S. aureus*	Unsorted	Stage 2 PJI	Stage 2 PJI

^*a*^ Most short group samples clustered as stage 1 PJI, while most long group samples clustered as stage 2 PJI. Intermediate group samples and unsorted samples clustered with both stage I and stage 2 PJI. NA, not applicable.

### Symptom duration proteomic findings do not predict DAIR surgical outcome

Proteins predictive of symptom duration were investigated in relation to DAIR failure. Hierarchical clustering of DAIR failure groups did not cluster samples by surgical outcome (Fig. 3C and D). While symptom duration is traditionally used to inform surgical management, here, symptom duration proteomics data did not predict DAIR surgical outcome.

### Demographics and common laboratory tests do not differentiate DAIR samples by surgical outcome

Whether patient demographics or commonly collected pre-surgical laboratory tests would inform DAIR failure was assessed. No tested metrics were significantly different between those with or without DAIR failure, including duration of symptoms, which is currently used to select the surgery type (Table 3).

**TABLE 3:** Demographic and pre-surgical laboratory data for the 21 patients who underwent DAIR surgery with known outcomes^*a*^

Parameter	DAIR success (*n* = 14)		DAIR failure (*n* = 7)	
	Mean	Standard deviation	Mean	Standard deviation
Number	14	–	7	–
Mean symptom duration (weeks)	2.57	2.59	4.69	4.38
Mean ESR (mm/hour)	60.85	34.83	47.29	24.55
Mean CRP (mg/dL)	118.19	73.70	125.27	133.06
Mean synovial fluid neutrophil (%)	93.18	5.91	81.86	24.31
Mean synovial fluid nucleated cell count (cells/mL)	65,990.64	41,375.86	51,856.86	87,633.25
Sinus tract present (%)	21.43	42.58	28.57	48.80
Female (%)	42.86	51.36	42.86	53.45
Mean age (years)	60.86	25.60	67.02	5.83
Leg: right (%)	42.86	42.58	42.86	48.80
Race: White (%)	92.86	26.73	100.00	0.00

^*a*^ There were no significant differences between groups with or without failure for any of the listed metrics (*P *> 0.05). LC-MS/MS was applied to 19/21 samples. ESR, erythrocyte sedimentation rate. CRP, C-reative protein.

### Multivariate analysis reveals “protein repair” as significantly functionally enriched in protein model predicting DAIR outcome

**Fig 4:**
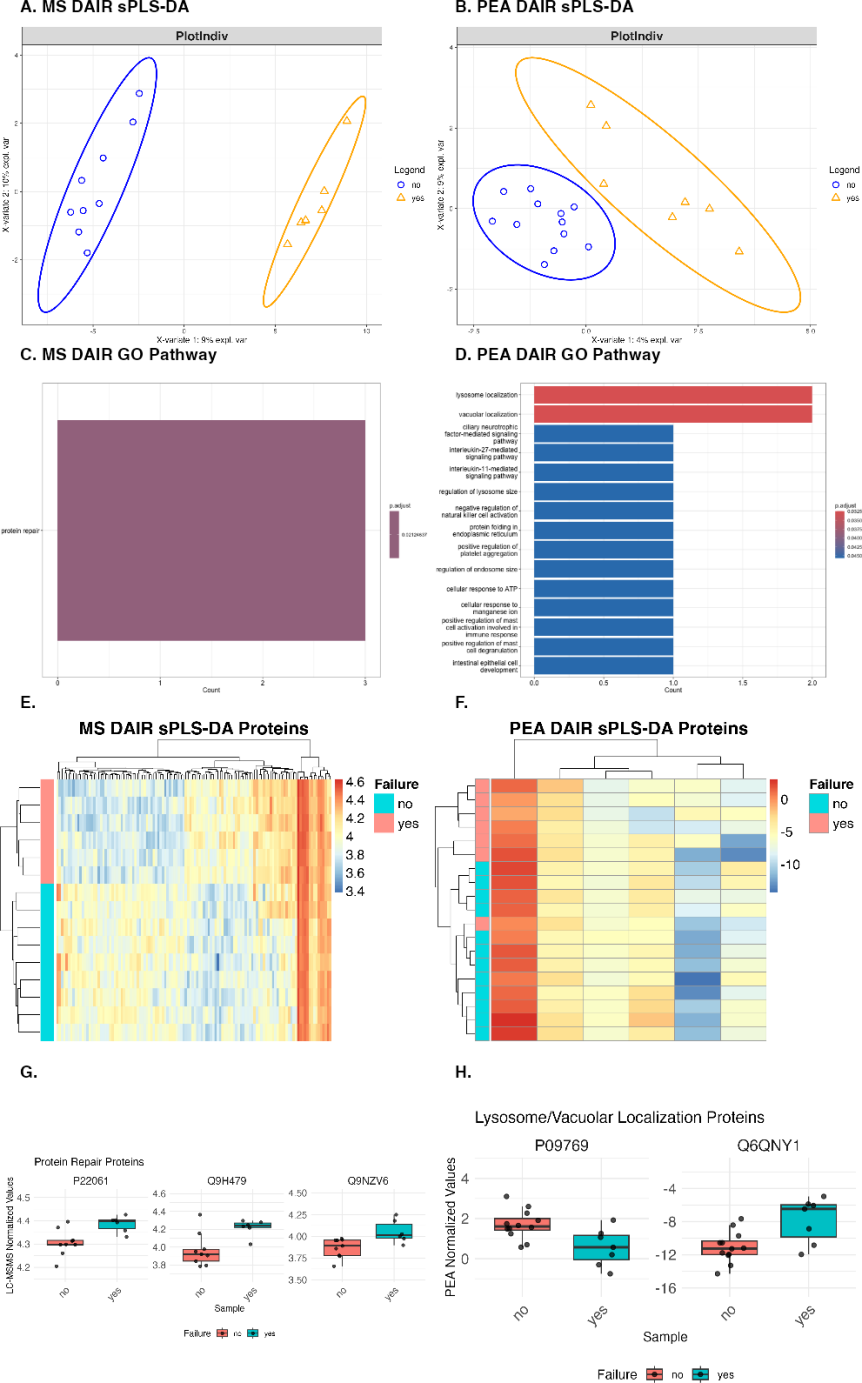
Multivariate analyses performed on LC-MS/MS and PEA data DAIR surgical outcomes. For both LC-MS/MS (**A**) and PEA (**B**), there was good separation between groups with and without failure. Although the mean AUC for the sPLS-DA model for PEA was lower than for LC-MS/MS (mean AUC PEA: 0.270247, mean AUC LC-MS/MS: 0.64073), LC-MS/MS had one significantly enriched GO pathway—“protein repair” (**C**); lysosome localization and vacuole localization were the most significant functionally enriched protein classes for PEA (**D**). Hierarchical clustering was performed; sPLS-DA-identified proteins were displayed using heatmaps for the LC-MS/MS and PEA data sets. LC-MS/MS data were separated into two primary groups by hierarchical clustering, with one containing all failure samples and the other containing only non-failure samples (**E**). In addition to hierarchical clustering, the heatmap showed a clear delineation between the groups with and without failure. With PEA data, visual delineation between groups was unclear, and one failure sample clustered with the no-failure samples (**F**). For LC-MS/MS, there were three proteins contributing to the protein repair functional group, P22061(PCMT1), Q9H479(FN3K), and Q9NZV6(MSRB1); all were elevated in the DAIR failure group, albeit not significantly (**G**). For PEA, there were two proteins contributing to lysosome localization and vacuole localization (**H**); one was upregulated (Q6QNY1/BLOC1S2) in the DAIR failure group, while the other was downregulated (P09769/FGR).

No proteins were significantly different between those with or without DAIR failure through univariate analysis (t-test with FDR adjustment). Multivariate analyses were then performed using sPLS-DA to sort “yes” failure (failed DAIR) and “no” failure (successful DAIR) groups and found clean sorting by LC-MS/MS along component 1, and by PEA along the diagonal of components 1 and 2 (Fig. 4A and B). One hundred twenty proteins were important for component 1 for LC-MS/MS, and six proteins across components 1 and 2 for PEA analysis. LC-MS/MS identified one functional group, “protein repair,” as functionally enriched in the sPLS-DA model for DAIR failure prediction (Fig. 4C; Table 4). PEA identified many functionally enriched pathways, most significantly lysosomal and vacuolar localization (Fig. 4D; Table 5). The LC-MS/MS model had a higher mean AUC (0.64) than the PEA (0.27) model. By hierarchical clustering, LC-MS/MS failure groups clustered based on surgical outcome (Fig. 4D). PEA correctly sorted all groups by surgical outcome, aside from one “yes” sample, which clustered with the “no” samples (Fig. 4E). The LC-MS/MS sPLS-DA model was functionally enriched for protein repair, including P22061(PCMT1), Q9H479(FN3K), and Q9NZV6(MSRB1); all three were upregulated in failed DAIR samples compared to successful samples (Fig. 4G). Two proteins were co-categorized as lysosomal/vacuolar localization proteins, categories functionally enriched in the PEA sPLS-DA model; however, they were not consistently up-regulated in the DAIR failure group (Fig. 4H).

**TABLE 4:** One hundred twenty sPLS-DA-identified proteins contributing to component 1 of the LC-MS/MS model predicting DAIR failure^*a*^

Protein	sPLS-DA weight	Gene	Protein	sPLS-DA weight	Gene
A0A075B6K0	−0.07	IGLV3-16	Q11201	−0.19	ST3GAL1
A0A075B6S6	−0.02	IGKV2D-30	Q14008	−0.01	CKAP5
O00329	0.07	PIK3CD	Q14974	0.03	KPNB1
O00442	0.09	RTCA	Q14BN4	−0.10	SLMAP
O00585	−0.05	CCL21	Q15181	0.02	PPA1
O14737	−0.15	PDCD5	Q15750	0.18	TAB1
O15067	0.11	PFAS	Q16394	−0.17	EXT1
O60684	0.05	KPNA6	Q4VC31	−0.02	MIX23
O60826	0.04	CCDC22	Q5SW79	−0.16	CEP170
O75443	−0.04	TECTA	Q68CZ2	−0.06	TNS3
O75663	0.08	TIPRL	Q6IAA8	0.05	LAMTOR1
O75711	0.00	SCRG1	Q6NYC8	0.00	PPP1R18
O76003	0.06	GLRX3	Q6Y7W6	−0.06	GIGYF2
O94855	0.06	SEC24D	Q7L1Q6	0.08	BZW1
O94906	−0.06	PRPF6	Q7L5D6	0.10	GET4
O95757	−0.21	HSPA4L	Q7Z7H8	−0.06	MRPL10
P00558	0.05	PGK1	Q8IWY4	−0.13	SCUBE1
P02775	0.06	PPBP	Q8N3C0	0.03	ASCC3
P04733	−0.02	MT1F	Q8TED0	−0.17	UTP15
P06865	0.05	HEXA	Q8WU76	−0.04	SCFD2
P07305	−0.03	H1-0	Q8WW59	−0.13	SPRYD4
P07311	0.11	ACYP1	Q92522	0.00	H1-10
P07814	0.12	EPRS1	Q92539	−0.12	LPIN2
P11684	−0.11	SCGB1A1	Q92581	0.15	SLC9A6
P12829	0.07	MYL4	Q96AP7	−0.11	ESAM
P15170	0.00	GSPT1	Q96B54	−0.04	ZNF428
P20138	−0.07	CD33	Q96BX8	0.04	MOB3A
P22061	0.06	PCMT1	Q96BY9	−0.13	SARAF
P22830	0.17	FECH	Q96C11	0.11	FGGY
P23490	−0.06	LORICRIN	Q96GG9	0.04	DCUN1D1
P23526	0.09	AHCY	Q96HD1	0.00	CRELD1
P24386	0.20	CHM	Q96JZ2	0.04	HSH2D
P24534	0.05	EEF1B2	Q96KN2	0.07	CNDP1
P24928	0.12	POLR2A	Q96MM6	−0.10	HSPA12B
P28161	0.20	GSTM2	Q96PZ2	−0.02	FAM111A
P30041	0.14	PRDX6	Q96RP7	−0.02	GAL3ST4
P33897	−0.05	ABCD1	Q9BVT8	−0.01	TMUB1
P33992	0.01	MCM5	Q9BY49	−0.01	PECR
P35443	−0.06	THBS4	Q9BY67	0.00	CADM1
P40123	−0.16	CAP2	Q9H009	−0.12	NACA2
P41250	0.03	GARS1	Q9H173	0.03	SIL1
P42345	−0.01	MTOR	Q9H300	−0.22	PARL
P45985	0.02	MAP2K4	Q9H479	0.06	FN3K
P47224	−0.01	RABIF	Q9HCJ6	0.00	VAT1L
P48147	0.02	PREP	Q9NPD3	0.00	EXOSC4
P49736	0.06	MCM2	Q9NUM4	0.03	TMEM106B
P49746	−0.04	THBS3	Q9NZV6	0.05	MSRB1
P50613	−0.18	CDK7	Q9P032	0.14	NDUFAF4
P52789	0.15	HK2	Q9P2J5	0.14	LARS1
P52888	0.02	THOP1	Q9UHD9	0.01	UBQLN2
P54886	0.05	ALDH18A1	Q9UI26	0.08	IPO11
P55036	0.07	PSMD4	Q9UIK4	0.06	DAPK2
P55072	0.18	VCP	Q9UKX2	−0.08	MYH2
P59768	−0.01	GNG2	Q9ULA0	−0.14	DNPEP
P60174	0.05	TPI1	Q9UNX4	−0.08	WDR3
P62913	−0.10	RPL11	Q9UPU7	0.09	TBC1D2B
P78417	0.00	GSTO1	Q9Y2D5	−0.03	PALM2AKAP2
P82675	0.00	MRPS5	Q9Y3C8	0.06	UFC1
Q01804	0.02	OTUD4	Q9Y487	−0.01	ATP6V0A2
Q0JRZ9	−0.11	FCHO2	Q9Y6B6	0.06	SAR1B

^*a*^ Uniprot and general consensus names are shown, along with the sPLS-DA calculated weight of each protein for component 1.

**TABLE 5:** Six unique sPLS-DA-identified proteins contributing to components 1 and 2 of the PEA model predicting DAIR failure^*a*^

Protein	sPLS-DA weight	Assay
O00534	−0.18	VWA5A
O00534	−1.00	VWA5A
P09769	−0.11	FGR
P14625	0.22	HSP90B1
P40189	−0.70	IL6ST
Q6QNY1	0.26	BLOC1S2
Q96PU4	−0.59	UHRF2

^*a*^ Uniprot and general consensus names are shown, along with the sPLS-DA calculated weight for proteins contributing to components 1 and 2. Notably, protein O00534 (von Willebrand factor A domain-containing protein 5A) contributed to both components 1 and 2.

## DISCUSSION

For surgeons and patients alike, DAIR is preferred over resection arthroplasty due to reduced morbidity and cost; yet its success rate is variable and difficult to predict. Oversimplification of PJI disease states as “acute” or “chronic” based on symptom duration may contribute to the reported range of DAIR success (25–35). An alternative approach would be to use a more objective predictor of DAIR outcome. Here, protein profiles associated with “DAIR-treatable” and “DAIR-untreatable” PJI, and short and long-duration PJI were investigated. Complementary LC-MS/MS and PEA approaches were used to analyze 7,261 unique human proteins in sonicate fluid. A group of 120 LC-MS/MS proteins, functionally enriched for protein repair, was identified that sorted samples by DAIR surgical outcome. Hierarchical sorting based on proteomics data primarily categorized two groups: “stage 1” and “stage 2” phases of infection, with a disease stage transition occurring between 4 and 12 weeks of symptoms, and Gene Ontology (GO) functional enrichment revealed distinct physiologies for PJI by symptom duration. In addition to a proteomics approach, commonly collected laboratory values and patient variables were queried, revealing that while CRP varied between symptom duration groups, no single commonly measured variable predicted DAIR outcome. Where traditional laboratory tests and symptom duration failed to predict DAIR failure, a multivariate proteomics approach sorted samples post hoc by surgical outcome, paving the way for potential exploration of a pre-surgical protein panel to guide surgical indications to select patients for DAIR.

Complementary techniques, LC-MS/MS and PEA, were used to survey over 7,000 human proteins in the sonicate fluid; each technique has strengths and weaknesses. The PEA and LC-MS/MS panels detected ~3,000 and ~5,000 proteins, respectively; the two technologies overlapped by 1,421 proteins. PEA technology can detect small proteins in minute quantities that might be missed by LC-MS/MS. However, PEA only assays on-panel proteins. LC-MS/MS may miss proteins present at low abundance, but can capture a larger range of proteins because it is hypothesis-free. Additionally, while not analyzed here, LC-MS/MS can identify microbial proteins. Given these differences, it is not surprising that the proteins identified as differentially abundant between samples differed. These differences were also observed in the GO pathways determined to be differentially enriched between samples. When determining differences between “stage 1” and “stage 2” PJI, both technologies were informative. However, when determining DAIR failure signatures, the LC-MS/MS sPLS-DA model was ultimately more informative. Despite this, for future studies, both methods should be considered to assess the full spectrum of proteins in human infections.

Studies of physiological differences between wounds that resolve in the acute phase or progress to a chronic phase may provide insight into DAIR outcome prediction. Non-healing acute wounds become chronic when they fail to make a key transition from an inflammatory to a proliferative phase of wound healing. Transition out of the former is mediated in part by efferocytosis—phagocytosis of apoptotic neutrophils by macrophages, leading to a transition from pro-inflammatory M1 to anti-inflammatory M2 macrophages (44–47). Beyond remaining arrested in the inflammatory phase of wound healing, compared to acute wounds, chronic wounds have increased elastase and matrix metalloproteinases (specifically MMP-9) (48, 49), decreased tissue inhibitors of metalloproteinase (TIMPs), decreased neutrophil apoptosis (50), increased neutrophil extracellular trap formation (NETosis) (51, 52), and persistence of macrophages in the M1-like pro-inflammatory phase. These changes prevent wound healing through runaway inflammation, leading to degradation of extracellular growth factors and the extracellular matrix necessary for healing (53, 54). There are similarities between the reported immunology of PJI and chronic wounds, such as enrichment of elastase (55), MMP-9 (56), neutrophils (57), NETosis (58, 59), and a decrease in M2 macrophages (60). Macrophage activation stage is now known to be broader than can be defined by classical “M1” and “M2” terminology, underscoring a need to study macrophage activation in complex infections such as PJI (61). Proteins predicting DAIR treatment were functionally enriched for protein repair, which may be related to protein destruction caused by MMP abundance and dysfunctional neutrophil and macrophage activity present in both PJI and chronic wounds. Future studies could investigate the variation in macrophage phase, the apoptotic and NETosis activities of neutrophils, the concentrations of MMPs and TIMPs in PJI, and their correlation with DAIR outcomes. Just as investigation into acute and chronic/non-healing wounds provides leads for therapies to improve wound resolution, further insight into similar markers may provide novel routes for the treatment of “DAIR-untreatable” PJI.

While disease “stage” sorting did not predict DAIR surgical outcome, there are benefits to elucidating the physiology of different infection stages. PEA identified factors normally associated with “acute” or “early” immune response to be upregulated in the long symptom duration group, including the humoral immune response, blood coagulation, complement activation, and response to interferon-γ and interleukin-1; this suggests possible delayed immune response to infection. Notably, multiple functional groups related to downregulated angiogenesis—the development of new blood vessels—were enriched in the short compared to the long-intermediate PJI groups. In wounds, angiogenesis is controlled by pro-angiogenic factors and angiogenesis inhibitory factors, initiated immediately after tissue injury, and is key for tissue repair. That angiogenesis is dysregulated in chronic wounds (62) suggests that a similar situation may occur in PJI. In one study, increasing angiogenesis in prosthetic vascular grafts via fibroblast growth factor decreased infections following aortic surgery (63). If dysfunctional angiogenesis is indeed related to PJI, it is plausible that similar interventions could decrease PJI rates or improve PJI outcomes (64). Undefined aspects of PJI host physiology may have hindered advancement and innovation in clinical treatment. Results presented suggest that delays in key immune responses, such as complement and humoral responses, as well as downregulation of angiogenesis in short-duration PJI, may play a role in PJI pathogenesis. Future studies focused on these aspects of immune response may inform future PJI prevention and treatment strategies.

There are several limitations of this work. First, the study relied on patient-reported symptom duration, a subjective measure of true infection length. The subjective nature of this category only further underscores the importance of using advanced omics approaches to classify PJI, rather than relying on patient-reported symptom duration. Also, classification of PJI is imperfect, and therefore, it is possible that some samples clinically diagnosed as having PJI were cases of non-infectious arthroplasty failure erroneously diagnosed as PJI. Sonicate fluid is generated by sonicating resected arthroplasty components such that the information it provides is only available after surgery; an ideal sample would be collected before surgery. This type of study could be repeated using synovial fluid, which can be collected before surgery. Additionally, PEA and LC-MS/MS provide relative abundance data, whereas clinical tests usually rely on absolute concentrations by volume. A final limitation is a lack of racial diversity.

In summary, an investigation of 7,261 unique human proteins separated sonicate fluids derived from PJI based on symptom duration and identified a proteomic profile predictive of DAIR success. The proteomic results presented lay the groundwork for transitioning away from broad categories of “acute” and “chronic” PJI toward using physiological markers to assess PJI disease states.

## MATERIALS AND METHODS

LC-MS/MS and PEA proteomics methodology are detailed in the Supplemental Methods. Sonicate fluids were collected and analyzed, and medical records reviewed with approval of the Mayo Clinic Institutional Review Board (#09-00808).

### Sample preparation

Explanted arthroplasty components were placed in autoclaved 1-L polypropylene wide-mouth containers in the operating room. Sterile Ringer’s solution (400 mL) was added to the container and vortexed (30 seconds), sonicated (40 kHz, 5 minutes), and vortexed (30 seconds). Sonicate fluid remained unconcentrated or was concentrated 100-fold by centrifugation at 3,150 × *g* for 5 minutes in conical centrifuge tubes, following which the supernatant was aspirated. Aliquots of unconcentrated and concentrated sonicated fluid were immediately frozen at −80°C.

### Sample handling: annotation and sorting

Ninety-five unconcentrated sonicated fluid samples underwent PEA analysis. A total of 77/95 sonicated fluid samples were submitted to LC-MS/MS analysis, with concentrated samples tested. Laboratory, demographic, and symptom duration data were collected for all PJI samples. Symptom duration data were unavailable for 8/95 (13.7%) samples (labeled as “unsorted” for heatmap analysis and excluded from *t*-test, Spearman, and sPLS-DA analyses).

### Data assembly

To calculate the percentage of female subjects, labels of M = 0 and F = 1 were applied, and the mean value was multiplied by 100. The same process was used to calculate the percentage of White (Not Hispanic or Latino) subjects, where White (Not Hispanic or Latino) subjects were labeled as 1, and other races (including African American, Hispanic, or Native American) as 0. When calculating sinus tract presence percentage, the same strategy was applied, with sinus tract presence as 1 and absence as 0. The same process was applied to calculate the percentage of right-sided knee surgeries, by labeling R = 1 and L = 0. To calculate mean CRP, “<3” values were converted to “2.9 mg/dL,” rather than omitting data. Age was calculated by subtracting the number of days between the surgical date and the date of birth and dividing the number by 365.25. Data were not manipulated to calculate means for synovial fluid neutrophil percentage, synovial fluid cell counts, or peripheral ESR (mm/h). Missing values were ignored. A standard *t*-test was used to determine *P* values for Tables 1 and 2.

### PEA normalization

Concentration-adjusted protein expression values were calculated based on raw read counts, extension control counts (internal control spike-in), and sample protein concentrations. Briefly, the raw read count for each sample per assay was divided by its respective extension control count to reach initial normalized protein expression values. Relative “c” values for each sample were determined, where c is the ratio of each sample’s protein concentration relative to the mean protein concentration of all samples. The initialized protein expression value for each sample per assay was multiplied by its respective “c” value to generate relative abundance data. Quantile normalization was performed using the normalize.quantiles function of PreProcessCore, and values transformed by log2 to create the final concentration-adjusted protein expression value.

### LC-MS/MS normalization

To normalize LC-MS/MS data, 0 values were replaced using “minprob” with a q = 0.01 in the DEP R package. Quantile normalization was performed, and data were log2 transformed.

### Univariate analysis: t-test and cutpointr

Using the t.test function in ggpubr, the *P* value between groups was calculated, and FDR values were generated from *P* values using the *P*.adjust function. All samples with an FDR ≤0.05 were run through cutpointr.

### Multivariate analysis: sPLS-DA

Using the tune and perf functions in the mixOmics package, an sPLS-DA model was tuned on the groups; if tuning selected an ncomp of 1, an ncomp of 2 was used, as that is a minimum requirement of the program. The perf command with “Mfold” validation was used to determine the mean AUC of the model.

### Generation of heatmaps and volcano plots

The pheatmap package was used to generate heatmaps of significant or important proteins with cluster_rows and cluster_cols set to TRUE. Volcano plots were created using the ggplot and geom_point functions in the ggplot2 package.

### Functional protein clustering

clusterProfiler and org.Hs.eg.db (database of human proteins) packages were used to cluster proteins by function and to generate figures. The enricher function was used to determine GO pathways.

## Data Availability

The mass spectrometry proteomics data have been deposited in the Proteome Xchange Consortium via the PRIDE partner repository with the data set identifier PXD058084. Analyses were run using R version 4.3.2 on an aarch64-apple-darwin20 platform. Code is available at https://github.com/RPatelLab/SonicateProteomics.

## SUPPLEMENTAL MATERIAL

Figure S1 mbio.01700-25-s0001.png Overlapping proteins and associated patterns between LC-MS/MS and PEA.

Figure S2 mbio.01700-25-s0002.tiff Upset plots showing protein overlap between GO pathways.

Figure S3 mbio.01700-25-s0003.tiff Functionally enriched KEGG pathways for short and long symptom duration PJI.

Supplemental material mbio.01700-25-s0004.docx Supplemental figure legends and methods.

Table S1 mbio.01700-25-s0005.csv Significant LC-MS/MS proteins between short and long symptom duration groups.

Table S2 mbio.01700-25-s0006.csv Significant PEA proteins identified by Spearman analysis.

Table S3 mbio.01700-25-s0007.csv Significant LC-MS/MS proteins identified by Spearman analysis.

Table S4 mbio.01700-25-s0008.csv sPLS-DA-identified LC-MS/MS proteins that differentiate short from long symptom duration groups.

Table S5 mbio.01700-25-s0009.csv sPLS-DA-identified PEA proteins that differentiate short from long symptom duration groups.

Table S6 mbio.01700-25-s0010.csv Significant PEA proteins that differentiate long-intermediate from short symptom duration groups.

ASM does not own the copyrights to Supplemental Material that may be linked to, or accessed through, an article. The authors have granted ASM a non-exclusive, world-wide license to publish the Supplemental Material files. Please contact the corresponding author directly for reuse.
