# Supplementary figures and images for: Proteomic profile at the time of surgery correlates with disease stage and surgical outcome in periprosthetic joint infection
Source: mBio. 2025 Aug 28;16(10):e01700-25. doi: 10.1128/mbio.01700-25 (PMC12505968; doi:10.1128/mbio.01700-25)

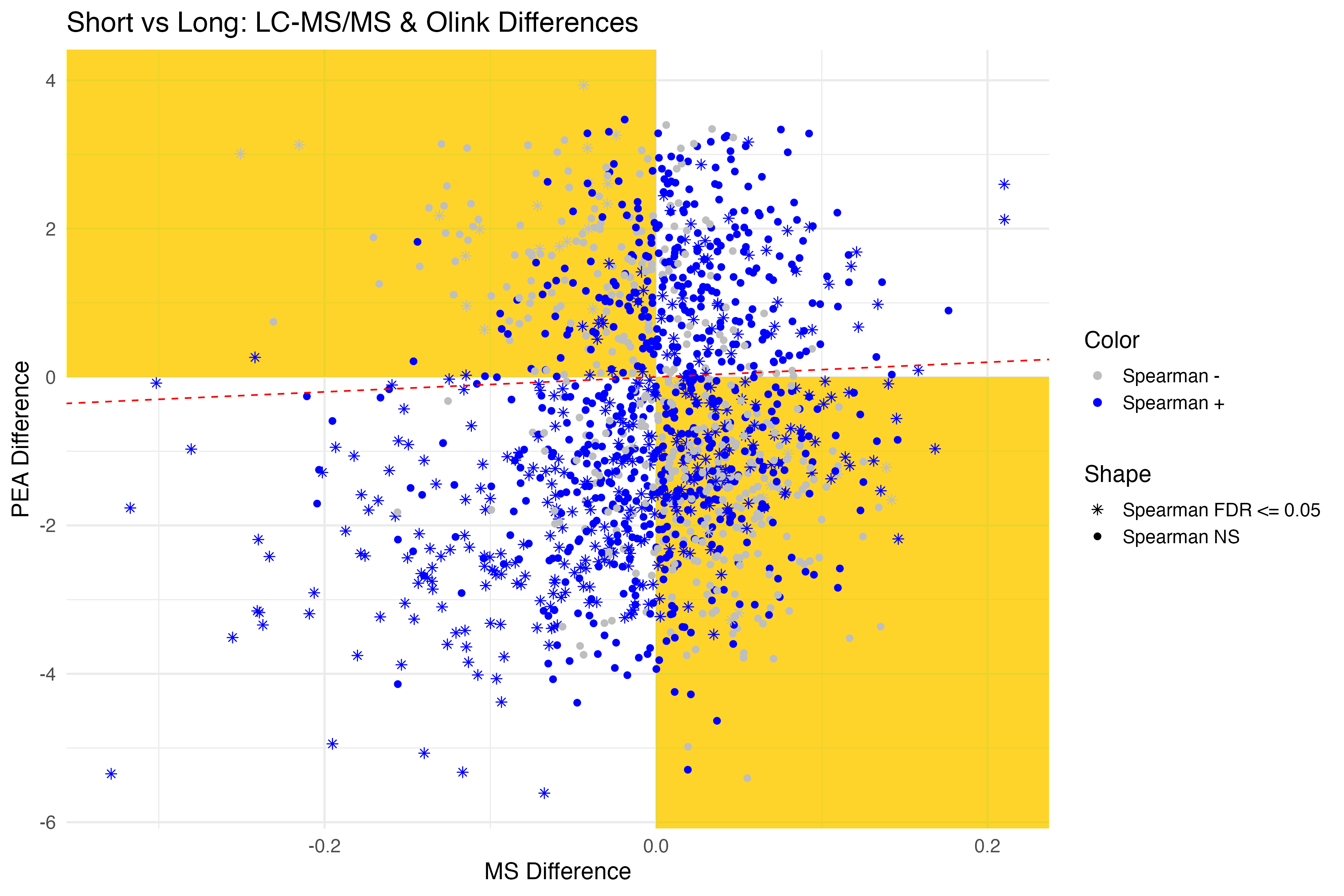

Supplement: Figure S1 — Overlapping proteins and associated patterns between LC-MS/MS and PEA. [file mbio.01700-25-s0001.png]

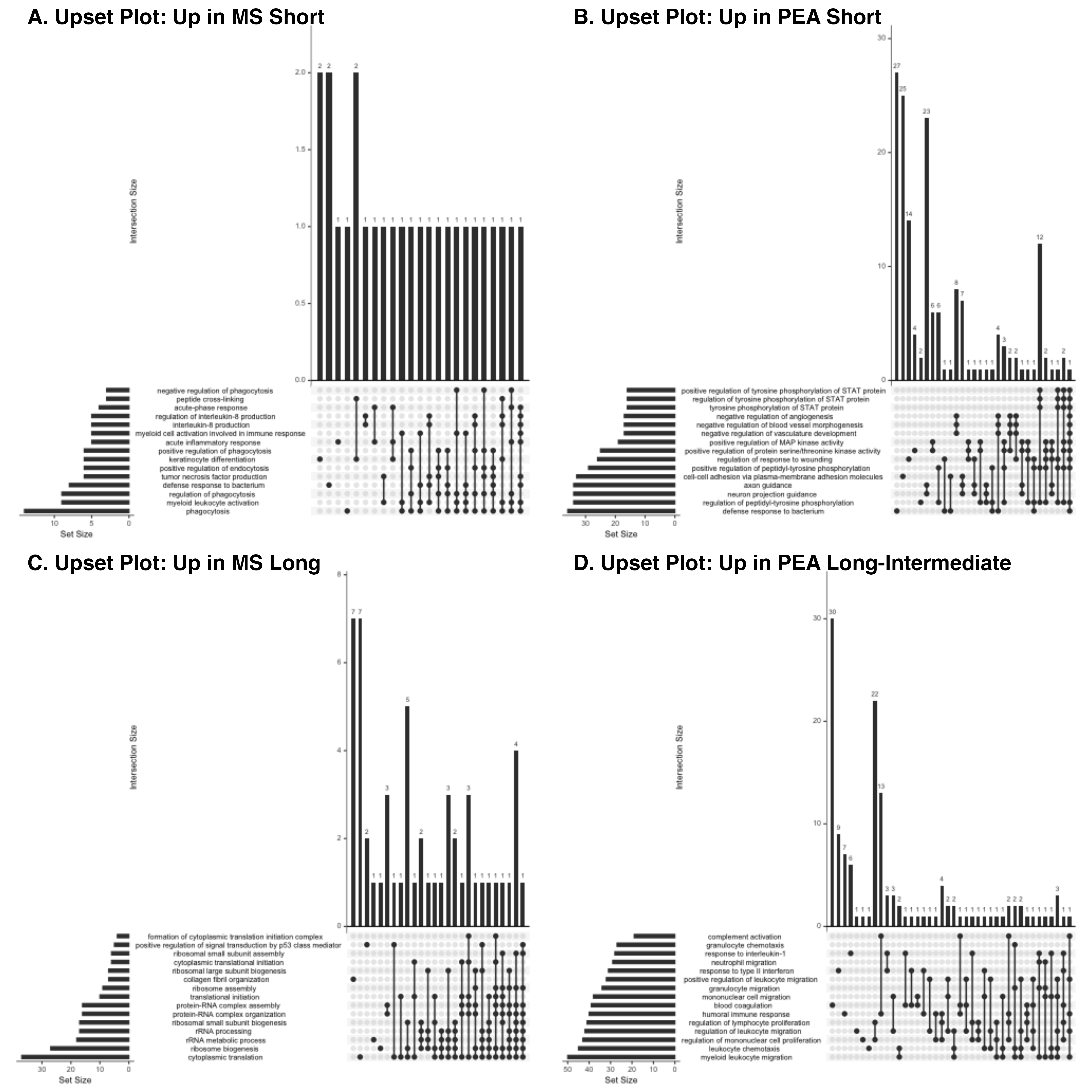

Supplement: Figure S2 — Upset plots showing protein overlap between GO pathways. [file mbio.01700-25-s0002.tiff]

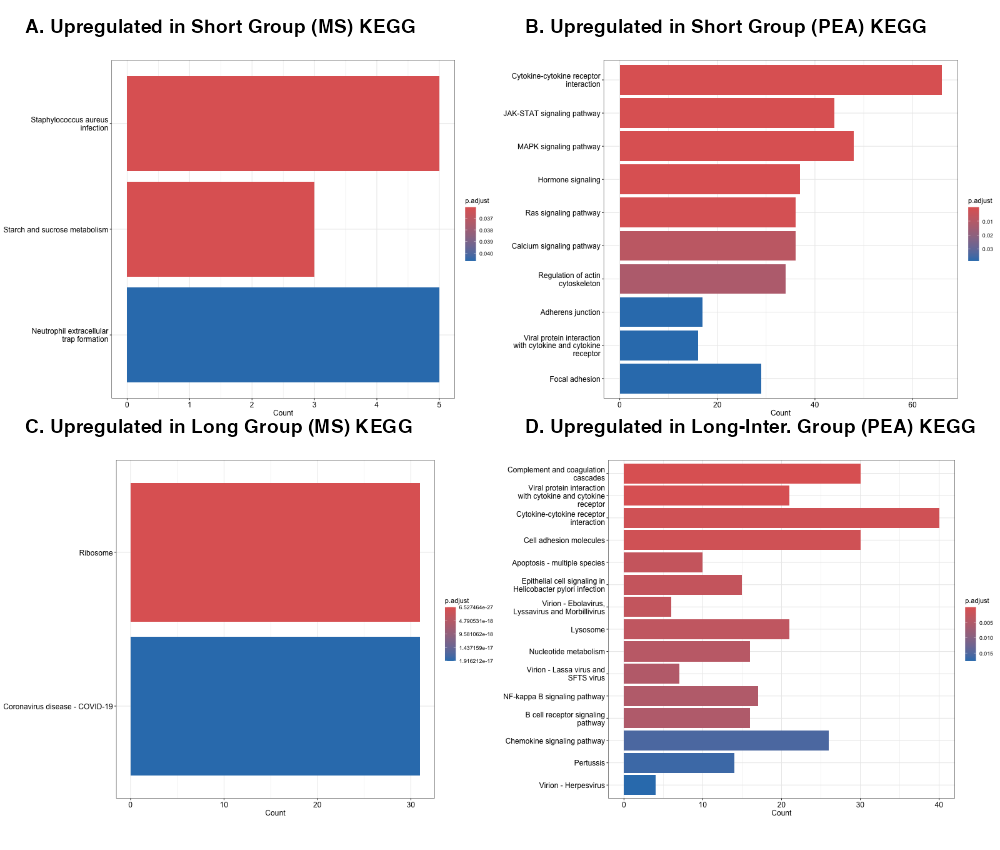

Supplement: Figure S3 — Functionally enriched KEGG pathways for short and long symptom duration PJI. [file mbio.01700-25-s0003.tiff]
