# Supplementary material for: Proteomic profile at the time of surgery correlates with disease stage and surgical outcome in periprosthetic joint infection
Source: mBio. 2025 Aug 28;16(10):e01700-25. doi: 10.1128/mbio.01700-25 (PMC12505968; doi:10.1128/mbio.01700-25)
Supplement: Supplemental material — Supplemental figure legends and methods. [file mbio.01700-25-s0004.docx]

Supplementary Methods

Sonicate Fluid Sample Processing for LC-MS/MS

Sonicate fluid samples were frozen at -80°C until processed. 300 µl of each sample was mixed with 20% sodium dodecyl sulfate (SDS) to a final concentration of 1% and then supplemented with 1X HALT protease/phosphatase inhibitor (Fisher Scientific, Waltham, MA). Samples were vortexed for 20 sec and subjected to probe sonication at 15% amplitude for 30 sec on ice. Samples were then centrifuged for 5 min at 15,000 x g. Supernatant was transferred to a 1.5 ml tube and protein estimation performed using BCA protein assay (Fisher Scientific). 100 µg of protein was dried using a vacuum concentrator and resuspended in 5% SDS, 50 mM triethylammonium bicarbonate buffer, pH 8.5. Reduction and alkylation were performed using 5 mM dithiothreitol for 45 min and 20 mM iodoacetamide for 30 min. Trypsin digestion was performed in S-Trap 96-well Mini Plate (Protifi, Fairport NY) per manufacturer recommendations. Briefly, protein lysates were acidified and mixed with 90% methanol/100 mM TEABC buffer before it was transferred to S-trap 96-well plate. Centrifugation was performed at 2,000 x g for 2 min followed by two washes with 90% methanol/100 mM TEABC buffer. Trypsin digestion was performed overnight at 37°C and peptides collected into a new 96-well plate and dried using speed vac. Dried peptides were stored at -80°C until LC-MS/MS analysis.

LC-MS/MS Sequencing

LC-MS/MS analysis was performed on a timsTOF HT mass spectrometer (Bruker Daltonics, Bremen, Germany) connected to an UltiMate 3000 RSLCnano system (Thermo Scientific).65,66 Briefly, peptides were reconstituted in 0.2% formic acid, 0.1% trifluoroacetic acid, and 0.0001% Zwittergent 3-16 and trapped on a loading column (EXP2 stem trap, Optimize Technologies) followed by separation on a PepSep Extreme analytical column (25cm x 150 μm x 1.5 µm C18 column, Bruker). Peptides were separated at a 1 μl/min flow rate using a gradient of 2% mobile phase A (0.1% formic acid in water) to 40% mobile phase B (80% acetonitrile, 0.1% formic acid) in 45 min, followed by a 5 mi ramp to 90% B that was held for 5 min before re-equilibrating to gradient starting conditions. Peptides were introduced via the Captive Spray source with 20 µm id emitter and analysis performed in the PASEF DDA mode where eluting peptides were accumulated in the first stage of the two-stage TIMS device before scanning out peptides from the second stage over a 1/k0 range of 0.7 to 1.43 in 100ms. MS and MS/MS spectra were collected over a range of 100-1700 m/z. The collision energy was linearly increased from 20 eV (0.6 Vs/cm2) to 59 eV (1.6 Vs/cm2).

Protein identification and quantitation was performed using FragPipe software (version 21.1). Database searching was performed by MSFragger search engine (version 4.0) against human UniProt protein database and contaminant proteins (downloaded 08/02/2024). Default search parameters were used, such as precursor and fragment ion tolerance of 20 ppm, strict trypsin cleavage specificity with two missed cleavages allowed, peptide length of 7-50 aa and mass range of 500-5000 Daltons. Methionine oxidation and N-terminal acetylation were specified as variable modifications and cysteine carbamidomethylation as a specific modification. Peptide spectral matches were rescored using MSBooster algorithm and validated by Percolator. Finally, false discovery rate was controlled by Philosopher tool (version 5.1) using a sequential FDR approach with 1% protein-level FDR. IonQuant (version 1.10.12) was used for protein quantitation and normalization using the MaxLFQ method.

PEA: Olink Explore 3072 Platform

Analysis on the Olink Explore 3072 platform was performed by the Mayo Clinic Proteomics Core according to the manufacturer’s instructions, to measure levels of 2,926 unique proteins by proximity extension immunoassay (PEA) (Olink Proteomics, Uppsala, Sweden) in sonicate fluid. The dynamic range of proteins was calibrated against serum. Sonicate fluid samples were tested without dilution. The technology converted analyte proteins-of-interest into a DNA library with sample- and protein-specific unique amplified DNA sequenced on a NovaSeq 6000 SP flow cell (Illumina, San Diego). Raw sequencing data were demultiplexed by Olink’s ‘bcl2counts’ script. Quality control of read count data was accessed using the NPX Explore software (Olink). Raw read and extension control read counts were exported for further manual normalization by sample protein concentration assessment.

**Supplementary Figure 1:** **Overlapping proteins and associated patterns between LC-MS/MS and PEA.** Spearman rank correlation was calculated for the 1,421 proteins that overlapped between PEA and LC-MS/MS and expression values plotted based on the difference between short and long groups.

**Supplementary Figure 2: Upset plots showing protein overlap between GO pathways**: To reflect the overlap between GO pathways, Upset plots were used for LC-MS/MS and PEA datasets. Many of the proteins contributed to multiple pathways for LC-MS/MS proteins upregulated in the short symptom duration group (A). Most proteins contributed to only one GO pathway for PEA upregulated proteins in the short symptom duration group (B), although there were a few proteins contributing to multiple pathways. Similar to what was observed in the short duration group, LC-MS/MS proteins had a higher number of proteins overlapping between pathways than PEA did for long or long-intermediate symptom duration groups.

**Supplementary Figure 3: Functionally enriched KEGG pathways for short and long symptom duration PJI.** To complement GO pathway findings, KEGG pathway analysis was performed on proteins that were significantly differentially regulated between short and long (A, C; LC-MS/MS), or short and long-intermediate (B, D; PEA) groups. KEGG pathway analysis identified different pathways, albeit similar in function.
